# Treatment of Abscess of the Maxillary Sinus

**Published:** 1893-10

**Authors:** E. Lecaudey

**Affiliations:** Paris, France


### TREATMENT OF ABSCESS OF THE MAXILLARY SINUS.[1]

BY DR. E. LECAUDEY, PARIS, FRANCE.

For more than thirty years I have cured among my clients over sixty cases of abscess of the maxillary sinus.

At first I employed a tin tube of one and a half millimetres in diameter, cut at each extremity, and with bent points, which I would fix in the inferior opening of the sinus. Experience taught me the length of time required in such a treatment, and the difficulty with which a fistula would close up. Having been a long time studying the subject, I once read a thesis by Dr. Veillard, treating of anal fistula, in which was described treatment with zinc chloride. The idea then struck me to apply the treatment to fistula of the sinus, and I obtained most excellent results.

The cases that I have oftenest treated were caused by the second bicuspid, less frequently by the cuspid and first molar. The treatment has generally lasted from eight to twenty-one days, and has never lasted more than six weeks. My observation has taught me that the more one dispenses with the small tube the quicker the fistula will close up. After having performed the extraction I wash carefully with hydrogen peroxide the cavity of the sinus. Then I inject the following:

---

[1] Read before the World's Columbian Dental Congress, Chicago, August, 1893.

Chloride of zinc, 1 gramme;
Phenic acid, 0.5 gramme;
Distilled water, 100 grammes.

In order to keep the fistula open, I place a little silk string, saturated with wax, and if, as rarely occurs, it will not close up, I take a small pencil of gutta-percha, which I saturate with a little chloride of zinc, place it for twenty-four hours inside of the fistula, and the edges close up by deep cicatrization very rapidly.

I have never had, owing to this method of treatment, any repetition of the disease.

